# Urinary Retention After AdVance™ XP Male Sling Placement Due to Herpes Zoster Virus Reactivation in the Immediate Postoperative Period: A Case Report

**DOI:** 10.7759/cureus.81070

**Published:** 2025-03-24

**Authors:** Merary Z Nazario-Perez, Valerie Rodriguez Baez, Rafael A Brito-Sanchez, Claudio Bernaschina-Bobadilla

**Affiliations:** 1 Urology, Saint Luke’s Hospital, Ponce, PRI; 2 Research Fellowship, Saint Luke’s Hospital, Ponce, PRI; 3 School of Medicine, Ponce Health Sciences University, Ponce, PRI; 4 Urology Residency Program, Saint Luke’s Hospital, Ponce, PRI

**Keywords:** acute urinary retention, advance™ xp male sling, herpes zoster virus, surgical stress, urinary stress incontinence, urological procedure

## Abstract

Herpes zoster virus, commonly known as shingles, results from the reactivation of the latent varicella-zoster virus (VZV) in the sensory ganglia and typically presents as painful, unilateral eruptions following a dermatomal distribution. While reactivation is often associated with aging, stress, malignancy, or immunosuppression, urinary retention (UR) due to bladder atony is a less common complication, particularly when the lumbosacral ganglia are affected. We present the case of a 63-year-old male who developed acute UR (AUR) following an AdVance™ XP Male Sling (Boston Scientific, Marlborough, Massachusetts, US) procedure for stress urinary incontinence (SUI). A workup, including cystoscopy and post-void residual (PVR) measurement, confirmed non-obstructive UR. Within days, the patient developed vesicular skin lesions over the L2, L4, S1, and S3 dermatomes, consistent with VZV reactivation. A multidisciplinary approach was implemented, and conservative management with antiviral therapy and catheterization led to complete symptom resolution. This is the first reported case of VZV reactivation following male sling placement, suggesting that surgical stress may be a trigger. Clinicians should recognize atypical VZV presentations, including UR, and prioritize conservative management before considering sling revision. Preoperative risk assessment, including vaccination for at-risk patients, may help prevent complications in future cases.

## Introduction

Voiding dysfunction (VD) is a recognized but uncommon complication of varicella-zoster virus (VZV) infection, with its reactivation, commonly known as shingles, affecting nearly 4% of patients. This condition arises from one of three inflammatory mechanisms: cystitis, myelitis, or neuritis, with neuritis being the most frequently implicated in bladder dysfunction [1]. In this process, VZV spreads from the dorsal root ganglia to the sacral motor neurons, causing inflammation that can lead to bladder atony, which is more common in patients with lumbosacral dermatome involvement [2]. VD, in these cases, often resolves as inflammation subsides.

The condition can be exacerbated by external triggers, such as surgical procedures, which induce significant physiological stress and represent an important risk factor for acute urinary retention (AUR) [3]. A particularly intriguing scenario arises when a patient with stress urinary incontinence (SUI) undergoes a continence procedure, such as a male sling procedure (MSP), only to develop postoperative UR. UR after MSP occurs in 12.9% of patients. The most frequently reported complications of male slings, from most to least common, include perineal pain (23%), explantation (0.9%), and infection (0.4%) [4-6]. Retention is typically temporary and resolves within several weeks; however, isolated cases have required sling release.

## Case presentation

A 63-year-old male presented for the evaluation of mild SUI after undergoing radical retropubic prostatectomy (RRP) in 2020 for the treatment of prostate cancer. He reported urinary leakage when coughing or during strenuous physical activity, using one to three pads per day. He denied dysuria, hematuria, polyuria, or urinary frequency. His medical history included Parkinson’s disease, depression, and genital herpes. The patient denied any toxic habits.

On physical examination, he appeared well-nourished and well-groomed, with stable vital signs and no signs of acute distress, fever, or tachycardia. After discussing treatment options, he opted for the placement of an AdVance™ XP Male Sling (Boston Scientific, Marlborough, Massachusetts, US). A preoperative cystoscopy revealed an open bladder neck, no strictures, adequate coaptation of the urethral sphincter, no inflammation, and mild USI. The surgery was performed without complications, and he was discharged in stable condition, voiding spontaneously. No skin lesions were observed before or during the MSP.

One week after surgery, the patient returned with urinary incontinence and mild pelvic pain. A pelvic ultrasound-guided post-void residual (PVR) measurement revealed a PVR of 700 mL. A Foley catheter was placed, draining 1000 mL, consistent with AUR. A voiding trial was attempted but was unsuccessful. A flexible cystoscopy ruled out mechanical obstruction of the sling, revealing a patent urethra, unobstructed bladder neck, normal-appearing bladder mucosa, and symmetric ureteral orifices with clear urine efflux. A Foley catheter was left in place, and naproxen 500 mg was prescribed for pain management.

**Figure 1:**
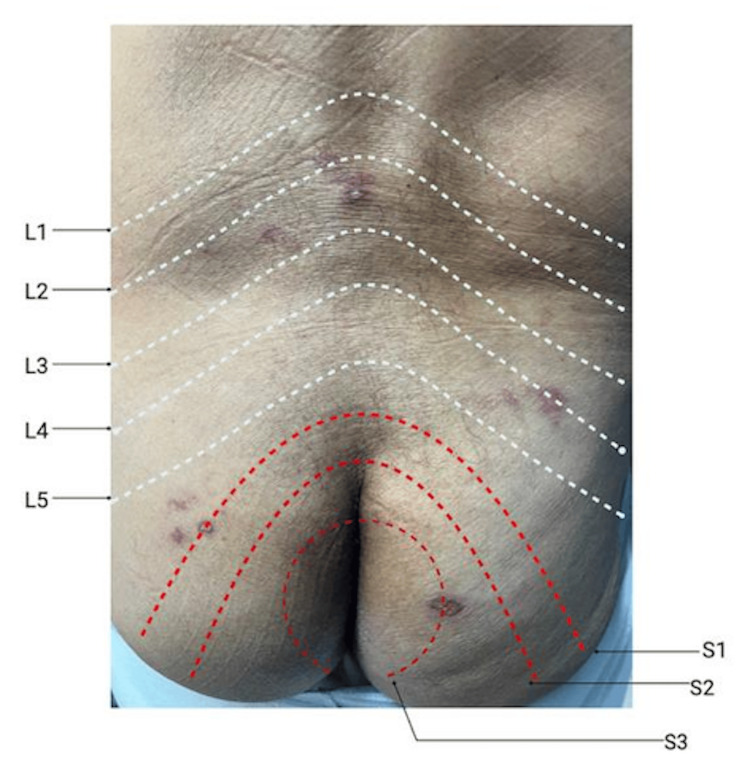
Healed vesicular lesions corresponding to lumbosacral dermatomes following AUR Multiple healed vesicular lesions were observed over the patient’s lower back and bilateral gluteal region, primarily affecting the L2, L4, S1, and S3 dermatomes. The lesions were identified following an episode of AUR and are consistent with varicella-zoster virus reactivation. AUR: acute urinary retention

During follow-up, the patient reported developing a rash on his lower back. Physical examination revealed healed vesicles primarily over the L2, L4, S1, and S3 dermatomes (Figure 1). The Foley catheter was removed; however, the patient failed the voiding trial. Given that the lesions were located in dermatomes associated with the pudendal and autonomic nerves, UR was attributed to bladder atony secondary to acute herpes zoster reactivation. Consequently, the patient was advised to receive the herpes zoster vaccine and was started on Neurontin and valacyclovir for symptomatic management.

The neurology service was consulted, and recommendations included maintaining the Foley catheter for 8-13 weeks, as this is the typical resolution time for VZV-associated UR. A repeat voiding trial was successful, with a PVR of 125 mL. Follow-up visits confirmed normal PVR and adequate urinary continence.

## Discussion

Our case report presents an uncommon instance of UR due to VZV reactivation in the immediate postoperative period following AdVance™ XP Male Sling placement. To our knowledge, this is the first documented case of AUR associated with VZV reactivation during the postoperative period of an AdVance™ XP Male Sling. The retention, in this case, was caused by neuritis rather than mechanical obstruction.

VZV infection can lead to UR through mechanisms such as myelitis, cystitis, or neuritis, all of which impair bladder function through inflammation and neural disruption [1]. Surgical stress induces neurohumoral and immunological responses that weaken immune defenses, allowing latent VZV to reactivate and disrupt neural pathways involved in bladder control. Factors such as tissue trauma, anesthesia, and postoperative recovery contribute to this immunosuppressive state. Less common presentations, such as VD, have also been reported after thoracic and cervical surgeries as well as shock wave lithotripsy [2,7,8].

Postoperative urinary retention (POUR) is a recognized complication of AdVance™ XP Male Sling, with studies reporting an incidence of 14.1% to 21.3% [1,2]. Initial management involves cystoscopic evaluation and transurethral bladder drainage for 24-48 hours, with suprapubic cystostomy considered if retention persists [4,5]. Persistent or late-onset retention warrants endoscopic and urodynamic evaluation to assess for mechanical obstruction, erosion, or detrusor failure [9]. For VZV-related UR, early antiviral therapy (within 72 hours) and non-steroidal anti-inflammatory drugs (NSAIDs) for pain management are recommended [10]. In our case, these steps were followed, and cystoscopy ruled out mechanical obstruction, highlighting the importance of a conservative approach before resorting to invasive surgical management.

Preoperative risk assessment is crucial, particularly in older patients with a prior history of VZV reactivation. Although vaccination provides well-documented benefits, its role as a standard component of preoperative risk assessment remains uncertain due to its low prevalence in the postoperative setting. The Centers for Disease Control and Prevention (CDC) recommends shingles vaccination for adults aged 50 years and older, with two doses administered two to six months apart. However, given the low incidence of VZV reactivation after surgery, routine vaccination screening may lack sufficient clinical justification in all surgical patients. Instead, selective vaccination strategies may be preferable in high-risk populations such as those with a history of recurrent VZV episodes or significant immunosuppression.

This case underscores the need for clinicians to increase the recognition of atypical VZV symptoms, including UR, pseudo-renal colic, and erectile dysfunction, particularly in patients with a history of trauma or surgical manipulation in the affected area [11]. A multidisciplinary approach remains essential for ensuring the timely diagnosis and management of VZV reactivation in surgical patients. Further research is warranted to explore the relationship between surgical stress and viral reactivation, as well as the potential benefits of preoperative vaccination in select patient populations.

## Conclusions

This case highlights the reactivation of VZV following a urological incontinence procedure, specifically presenting as UR after AdVance™ XP Male Sling placement. Surgical stress is a well-recognized trigger for various physiological responses, and its role in VZV reactivation warrants further investigation. To our knowledge, this is the first reported case of such an occurrence. Urologists should adopt a conservative approach, including cystoscopy, to rule out urethral obstruction while also recognizing that UR may have non-mechanical causes such as viral reactivation. This underscores the importance of considering VZV in the differential diagnosis of postoperative UR. Further research should explore the extent to which surgical stress contributes to VZV reactivation, particularly in older patients with a history of recurrence. Vaccination remains a crucial preventive measure for at-risk individuals undergoing surgery, and a multidisciplinary approach can help mitigate herpes zoster-related complications. Standardizing preoperative risk assessments and improving clinician and patient education may enhance early recognition of UR as a potential symptom of VZV reactivation, ensuring timely intervention and appropriate urological referral.
